# The Ordered Clustered Travelling Salesman Problem: A Hybrid Genetic Algorithm

**DOI:** 10.1155/2014/258207

**Published:** 2014-02-19

**Authors:** Zakir Hussain Ahmed

**Affiliations:** Department of Computer Science, Al Imam Mohammad Ibn Saud Islamic University (IMSIU), P.O. Box 5701, Riyadh 11432, Saudi Arabia

## Abstract

The ordered clustered travelling salesman problem is a variation of the usual travelling salesman problem in which a set of vertices (except the starting vertex) of the network is divided into some prespecified clusters. The objective is to find the least cost Hamiltonian tour in which vertices of any cluster are visited contiguously and the clusters are visited in the prespecified order. The problem is NP-hard, and it arises in practical transportation and sequencing problems. This paper develops a hybrid genetic algorithm using sequential constructive crossover, 2-opt search, and a local search for obtaining heuristic solution to the problem. The efficiency of the algorithm has been examined against two existing algorithms for some asymmetric and symmetric TSPLIB instances of various sizes. The computational results show that the proposed algorithm is very effective in terms of solution quality and computational time. Finally, we present solution to some more symmetric TSPLIB instances.

## 1. Introduction

The clustered travelling salesman problem (CTSP), introduced by Chisman [1], is a variation of the usual travelling salesman problem (TSP). It can be defined as follows: let *G* = (*V*, *E*) be a complete undirected graph with vertex set *V* and edge set *E*. The vertex set *V* = {*v*_1_, *v*_2_,…, *v*_*n*_}, except the starting vertex (depot) *v*_1_, is partitioned into *m* prespecified clusters *V*_1_, *V*_2_,…, *V*_*m*_. The number of vertices in the clusters (i.e., size of the clusters) is *n*_1_, *n*_2_,…, *n*_*m*_, respectively. A cost matrix *C* = [*c*_*ij*_] representing travel costs, distances, or travel times is defined on the edge set *E* = {(*v*_*i*_, *v*_*j*_) : *v*_*i*_,  *v*_*j*_ ∈ *V*,  *i* ≠ *j*}. Starting from the depot *v*_1_, the objective of the CTSP is to determine the least cost Hamiltonian tour on *G* in which the vertices of any cluster *V*_*k*_ are visited contiguously, and the clusters are visited in the order *V*_1_, *V*_2_,…, *V*_*m*_.

There are several variants of the problem depending on whether the start and end vertices of a cluster as well as the number and order of clusters have been specified. If the number of clusters is either one or each cluster has only one vertex, then the problem becomes the usual TSP. If the number of clusters is two then the problem is called TSP with backhauls (TSPB) [2]. In the free CTSP, the cluster order is not prespecified and the problem is to simultaneously determine the optimal cluster order as well as the routing within and between clusters. This paper focuses on the variant with specified order of clusters and unspecified end vertices of the clusters, which is called ordered CTSP (OCTSP). For simplicity, we label the vertices as natural numbers from 1 to *n* and, thus, assume that the label of the vertices of any cluster is less than the label of the vertices of the following clusters.

Since all the variations are generalization of the usual TSP, they all are NP-hard [3]. The CTSP has many applications in real life, for example, in automated warehouse routing [1, 4], in production planning [4], and in vehicle routing, manufacturing, computer operations, examination timetabling, cytological testing, and integrated circuit testing [5, 6]. Chisman [1] showed that the CTSP can be transformed into a TSP by adding or subtracting an arbitrarily large constant *M* to or from the cost of each intercluster edge. Therefore, after the transformation, any exact algorithm for the TSP can be applied to solve the problem exactly. However, as the size increases finding exact optimal solution to the CTSP instances becomes impractical, and hence, heuristic must be used.

We seek approximate solution using heuristic algorithm for the OCTSP. For the TSP and related problems, well-known heuristic algorithms are genetic algorithms (GAs), tabu search (TS), artificial neural network (ANN), simulated annealing (SA), approximate algorithms, and so forth. Amongst the heuristics, GAs are found to be the best algorithms for the TSP and its variations. Since OCTSP is a variation of the usual TSP, therefore, we develop a hybrid GA (HGA) using sequential constructive crossover [7] and 2-opt search and a local search [8] to obtain heuristically optimal solution to the problem. The efficiency of our algorithm has been examined against partitioning algorithm [9] for some medium sized asymmetric TSPLIB [10] instances and lexisearch algorithm [11] for some small sized symmetric TSPLIB [10] instances. The computational experiments show the effectiveness of our proposed HGA. Finally, we present solution to some medium sized symmetric TSPLIB [10] instances. However, to the best of our knowledge, no literature presents solution to these symmetric instances. Hence, we could not provide any comparative study of these results.

This paper is organized as follows.  Section 2 presents a detailed literature review to the problem. A hybrid genetic algorithm is developed and reported in Section 3. Computational experiment for the algorithm is presented in Section 4. Finally, Section 5 presents comments and concluding remarks.

## 2. Literature Review

Chisman [1] transformed the CTSP to the usual TSP and then applied branch and bound approach [12] to solve the problem exactly but did not obtain very good results. Thereafter, Lokin [4] and Jongens and Volgenant [13] applied exact algorithms to find exact optimal solution to the problem. Aramgiatisiris [9] developed an exact partitioning algorithm (LBDCOMP therein) by transforming the problem to the TSPB, then solving independently linehaul and backhaul subproblems, and finally reformulating as a direct shortest path on the bipartite graph problems. However, the algorithm does not really obtain exact solutions for many instances [11].

An approximation algorithm with good empirical performance [14] was developed to solve the problem with a prespecified order on the clusters. Also, three more heuristics were proposed and compared among them. As reported, the best results were obtained by the heuristic that first transforms the problem into a TSP and then applies the GENIUS heuristic.

Laporte et al. [5] proposed a TS heuristic that combined with a phase of diversification using a GA to solve the problem with a prespecified order of visiting the clusters. As reported, the TS outperforms the GA [15] that exploits order-based crossover operators and local search heuristics. However, when comparing TS with a postoptimization procedure [14], TS obtained better quality of solutions but required more computational time.

Another GA was developed for the problem [16] that first finds intercluster paths and then intracluster paths. Finally, a comparative study of the GA was presented against a GENIUS heuristic [14] and lower bounds [13]. As reported, the GA could solve instances up to 500 vertices with 4 and 10 clusters and obtained solutions within 5.5% of the lower bound.

An approximation algorithm of 5/3 performance ratio has been developed for the OCTSP with a prespecified visiting sequence for the clusters [17]. Another approximation algorithm has been proposed which guarantees bounded performance for some variants of the CTSP [3]. For the problem with unspecified end vertices, its algorithm first uses a modified Christofides' algorithm [18] to get the shortest free ends Hamiltonian paths in each cluster. After the first step, the two end vertices for each cluster and the intracluster paths are specified. Then a rural postman problem algorithm is used to connect all the intracluster paths to form a whole tour. This algorithm favours the intracluster Hamiltonian paths, which implies that the inter-cluster paths may be sacrificed when the end vertices in each cluster are already determined.

A two-level-TSP hierarchical algorithm that favours intercluster paths has been proposed for the CTSP [19]. First, the shortest intercluster paths connecting every cluster have been specified; then the start and end vertices are specified for each cluster. Next, a modified Christofides' algorithm [18] is used to get the shortest Hamiltonian paths with two specified end vertices in each cluster. At the end, a whole tour is formed by combining the paths generated in both levels. They also showed that the penalties caused by favoring the intracluster Hamiltonian paths and the intercluster paths are comparable.

A two-level GA (TLGA) has been developed for solving CTSP with unspecified end vertices [20]. The algorithm first finds the shortest Hamiltonian cycle for each cluster and then connects all the intracluster paths in a certain sequence to form a whole tour. In the lower level, a GA is used to find the shortest Hamiltonian cycle rather than the shortest Hamiltonian path for each cluster. In the higher level, a modified GA is designed to determine an edge that will be deleted from the shortest Hamiltonian cycle for each cluster and the visiting sequence of all the clusters with the objective of shortest travelling tour for the whole problem. The higher level algorithm has the freedom to delete any edge of the clusters while searching for the shortest complete tour. Test results demonstrate that the TLGA for large TSPs is more effective and efficient than the classical GA.

## 3. A Hybrid Genetic Algorithm for the OCTSP

### 3.1. A Brief Overview of GAs

GAs are structured, yet randomized, search methods based on mimicking the survival of the fittest among the species generated by random changes in the gene structure of the chromosomes in the evolutionary biology [21]. They start with a population of chromosomes (solutions) that evolve from one generation to the next. Each generation consists of the following three operations.

- *Selection*. This procedure is a stochastic process that mimics the “survival-of-fittest” theory. However, here no new chromosome is created. Some of the chromosomes are copied (even more than once) to the next generation probabilistically based on their objective function value, whereas some other chromosomes are discarded.
- *Crossover*. It is a binary operator that applies to two parent chromosomes with a large probability, which creates new offspring chromosome(s). It is a very important operator in GAs. Also, crossover operator together with selection operator is found to be the most powerful process in the GA search.
- *Mutation*. It is a unary operator that applies to each of the chromosomes with a small probability. It is the occasional random change of some selected gene(s) of a chromosome to diversify the GA search space.

Starting from a randomly generated or heuristically generated initial population, the GAs search repeated the above three operators until the stopping criterion is satisfied. Crossover operator is a unique feature of GAs that wishes to combine good quality parent chromosomes to create one or more new offspring chromosome(s). However, it is seen that the crossover alone cannot generate high quality chromosomes for the combinatorial optimization problems, like the TSP and its variations. Thus, powerful local search methods are incorporated to improve the quality of offspring chromosomes [5]. In hybrid GAs, crossover operator generates new starting solutions for the local search methods.

**Figure 1:**
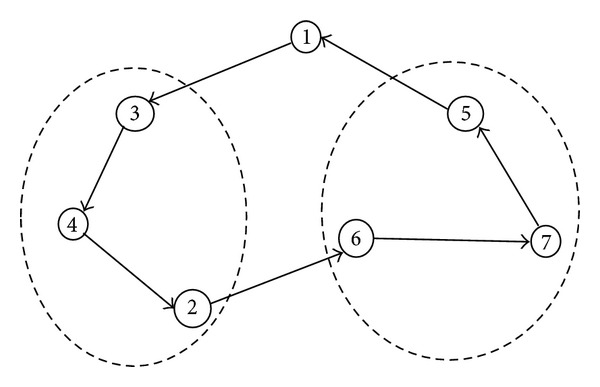
An example of result of the OCTSP using GA.

GAs are found to be successful heuristic algorithms for solving the usual TSP and its variations. However, GAs do not guarantee the optimality of the solution, but they can find very good, near optimal solution in very short time. We are applying crossover, mutation, and local search methods for each cluster in the prespecified order for the OCTSP. Result of the GA for a 7-vertex problem instance is a complete tour as shown in Figure 1.

### 3.2. Bias Removal

Bias removal step is adopted in lexisearch algorithm [11] and found effective for the CTSP. We also consider the bias removal step in our GA. The main advantage of the bias removal is that a large amount of the solution value is kept fixed, and for the remaining small value we have to search. The process for bias removal of the cost matrix is as follows: subtract each row-minima from its corresponding row elements, repeat the same column-wise on the resultant matrix. The total of the row-minima and the subsequent column-minima is called the “bias” of the matrix. However, we have not incorporated clusters precedence relations in our cost matrix. This does not affect the value of a chromosome, since, while generating a chromosome, the clusters precedence relations are taken care of.

The bias of the cost matrix given in Table 1 is (row-minima + column-minima = 129 + 19 =) 148. The reduced cost matrix (i.e., after removing bias of the matrix) is shown in Table 2. We shall solve the problem with respect to the reduced cost matrix. After we find solution value with respect to the reduced matrix, we shall add the bias to the value for finding the solution value with respect to the original cost matrix.

### 3.3. Alphabet Table

Alphabet matrix, *A* = [*a*(*i*, *j*)], is a square matrix of order *n* formed by positions of elements of the reduced cost matrix of order *n*, *C*′ = [*c*_*ij*_′], when they are arranged in the nondecreasing order of their costs. Alphabet table “[*a*(*i*, *j*) − *c*_*i*,*a*(*i*,*j*)_′]” is the combination of elements (vertices) of matrix A and their costs in the reduced matrix [11]. The alphabet table for the reduced cost matrix in Table 2 is shown in Table 3. This alphabet table also is not taking care of the clusters precedence relations.

### 3.4. Improved Initial Population

The path representation for a chromosome is used in our GA. In this representation, any vertex is assigned to a unique natural number from 1 to *n*; that is, genes are natural numbers. The path of a salesman is represented by a chromosome which is a permutation of number genes. A gene segment is defined as a permutation of the vertices in a cluster. A chromosome is a permutation of all the gene segments with one gene segment per cluster. For example, let {1,2, 3,4, 5,6, 7} be the vertices with *V*_1_ = {2,3, 4}, *V*_2_ = {5,6, 7}, and *V*_1_ is followed by *V*_2_, in a 7-vertex instance; then, starting from vertex 1, a complete tour {1→3→4→2→6→7→5→1} may be represented as the chromosome (1, 3, 4, 2, 6, 7, 5), where (3, 4, 2) and (6, 7, 5) are the gene segments for cluster 1 and cluster 2, respectively.

It is to be noted that starting from a good initial population can deliver better quality of solutions quickly, and that is why many literatures report generating initial population using heuristics. Hence, we are going to use sequential sampling algorithm for heuristically generating initial population that has been applied successfully to the bottleneck TSP [22]. This algorithm is a simple version of the sequential constructive sampling algorithm [8]. It is basically a probabilistic method to generate a tour of the salesman. The probability of visiting each unvisited vertex of a cluster in a row of the alphabet table is assigned in such a way that the first unvisited vertex gets more probability than the second one, and so on. Thereafter, cumulative probability of each unvisited vertex of a cluster is calculated. Next, a random number, *r* ∈ [0,1], is generated and the vertex that represents the chosen random number in the cumulative probability range is accepted. The probability of visiting each unvisited vertex of a cluster is assigned as follows. Suppose the number of unvisited vertices of a cluster in a row of the alphabet table is *k*. The probability of visiting the *i*th unvisited vertex is

(1) pi=2(k−i+1)k(k+1).

The algorithm may be summarized as follows.

**Step 0**

Construct the “alphabet table” based on the reduced cost matrix. Repeat the following steps for the fixed population size (*P*_*s*_).

**Step 1**

Since “vertex 1” is the starting vertex, so, initialize *p* = 1 and go to Step 2.

**Step 2**

Go to the *p*th row of the “alphabet table” and visit probabilistically, by using (1), any unvisited vertex of the row (say vertex *q*) in the present cluster and go to Step 3.

**Step 3**

Rename the “vertex *q*” as “vertex *p*” and go to Step 4.

**Step 4**

If all vertices of the present cluster are visited then go to the next cluster in the order (if any) and go to Step 5; else go to Step 2.

**Step 5**

If all vertices of the network are visited then go to Step 1 for generating another chromosome in the population; else go to Step 2.

Let us illustrate the algorithm through the example given in Table 1 with *V*_1_ = {2,3, 4}, *V*_2_ = {5,6, 7}, and *V*_1_ is followed by *V*_2_. We start from 1st row of the “alphabet table.” The number of unvisited vertices of the 1st cluster in the row is 3, which are 4, 2, and 3, with cumulative probabilities 0.500, 0.833, and 1.000, respectively. Suppose the vertex 4 is selected randomly; then the partial chromosome will be (1, 4). Next we go to 4th row of the “alphabet table” and probabilistically select another node, and so on. Proceeding in this way it may lead to a complete chromosome (1, 4, 2, 3, 6, 5, 7).

A preliminary study shows the effectiveness of the sampling algorithm for initial population. However, instead of considering all unvisited vertices if we consider at most first ten vertices in a cluster then the algorithm generates very good population. A similar observation has been reported for the bottleneck TSP also [22]. Hence, we consider this restricted domain of unvisited vertices of a cluster for our study. Further, to start with better population, we apply 2-opt search to each chromosome. The 2-opt search removes two edges and then replaces them by a different set of edges in such a way so as to maintain the feasibility of the tour. Let *α*_*i*_, *α*_*i*+1_, *α*_*j*_, and *α*_*j*+1_ be four vertices in a cluster; then if the edges (*α*_*i*_, *α*_*i*+1_) and (*α*_*j*_, *α*_*j*+1_) are removed, the only way to form a new valid tour is to connect *α*_*i*_ to *α*_*j*_ and *α*_*i*+1_ to *α*_*j*+1_.

### 3.5. Fitness Function and Selection Method

The objective function of each chromosome in the population is the cost of the tour represented by the chromosome. The fitness function of a chromosome is defined as multiplicative inverse of the objective function. There are various selection methods in the literature. The selection operation considered for our study is the stochastic remainder selection method [23].

### 3.6. The Sequential Constructive Crossover Operation

Since crossover operation plays main role in GAs, hence, several crossover operators have been proposed for the usual TSP, which are then used for the variant TSPs also. Out of them, the sequential constructive crossover (SCX) [7] is found to be one of the best crossover operators for the usual TSP. A multiparent extension of the SCX has been applied to the usual TSP and found good results [24]. The SCX has also been successfully applied to the TSP with some other variations [22, 25]. In general, it produces an offspring using better edges of the parents. However, it does not depend only on the parents' structure; it sometimes introduces new, but good, edges to the offspring, which are not even available in the present population. We modify the SCX operator for the OCTSP as follows.

**Step 1**

Start from “vertex 1” (i.e., current vertex *p* = 1).

**Step 2**

Sequentially search both of the parent chromosomes and consider the first unvisited vertex of the present cluster appearing after “vertex *p*” in each parent. If no unvisited vertex after “vertex *p*” is available in any (or both) of the parents, search sequentially from the starting of that parent and consider the first unvisited vertex of the cluster, and go to Step 3.

**Step 3**

Suppose the “vertex *α*” and the “vertex *β*” are found in the 1st and 2nd parents respectively, then for selecting the next vertex in the offspring chromosome go to Step 4.

**Step 4**

If *c*_*pα*_ < *c*_*pβ*_, then select “vertex *α*”; otherwise, select “vertex *β*” as the next vertex and concatenate it to the partially constructed offspring chromosome and go to Step 5.

**Step 5**

If there is not any vertex left in that cluster, then go to the next cluster, if any. If the offspring is a complete chromosome, then stop; otherwise, rename the present vertex as “vertex *p*” and go to Step 2.

**Figure 2:**
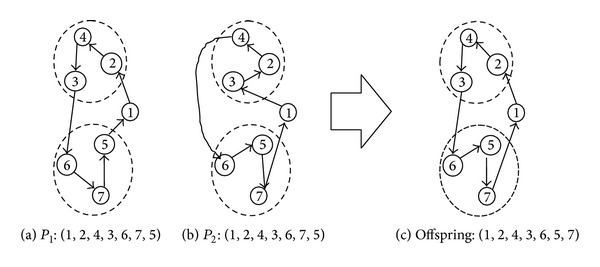
Example of sequential constructive crossover operation.

Let a pair of parent chromosomes be *P*_1_: (1, 2, 4, 3, 6, 7, 5) and *P*_2_: (1, 3, 2, 4, 6, 5, 7) with costs 357 and 354, respectively, with respect to the original cost matrix given in Table 1. By applying above SCX, we obtain the offspring chromosome (1, 2, 4, 3, 6, 5, 7) with cost 318, which is less than both parents. The parent and the offspring chromosomes are shown in Figure 2. In general, crossover operator inherits parents' characteristics, and the operator that preserves good characteristics of parents in the offspring is said to be good operator. The SCX is found to be excellent in this regard. Bold edges in Figure 2(c) are the edges which are available either in the first parent or in second parent. For this given example, all edges are selected from either of the parents.

For the crossover operation, a pair of parent chromosomes is selected sequentially from the mating pool. It is reported that the SCX gets stuck in local minimums quickly for the TSP [7], which is very often due to the identical population. So, to overcome this situation, the selected parents are checked for duplication. If the selected parents are found to be identical, then the second parent is modified temporarily by swapping some randomly chosen pair of genes in the chromosome, and then the crossover operation is performed. To improve quality of the solution as well as have a mixture of parents and offspring in a population, the first parent is replaced by the offspring only if the offspring value is better than the average value of the present population. In this way, the mixed population retains diversity also. To further improve the quality of the solution obtained by crossover, many researchers applied 2-opt search operator. To improve solution quality, we are going to use a local search method that combines three mutation operators that will be discussed in Section 3.8. However, we are not applying this local search method to all of the offspring; rather, it is applied only to the offspring if its value is better than the average population value. Now, since our crossover operator produces only one offspring, to keep population size fixed throughout the generations, while pairing with the next chromosome in order, the present second original parent chromosome will be considered as the first parent, and so on.

### 3.7. Mutation Operation

**Figure 3:**
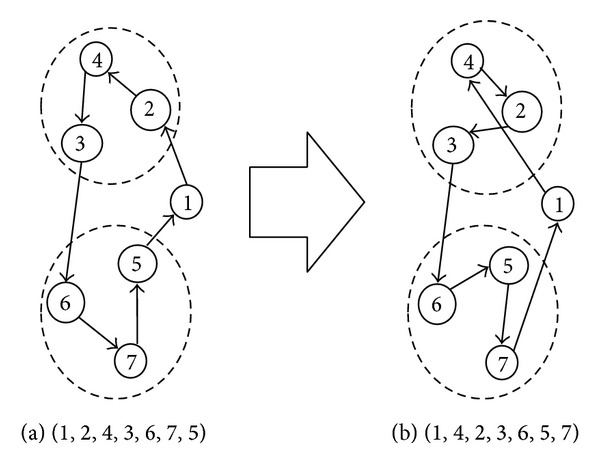
Example of reciprocal exchange mutation operation.

The mutation operator randomly selects a position in the chromosome and changes the corresponding gene, thereby modifying information. The need for mutation comes from the fact that, as the less fit chromosomes of successive generations are discarded, some aspects of genetic material could be lost forever. By performing occasional random changes in the chromosomes, GAs ensure that new parts of the search space are reached, which selection and crossover could not fully guarantee. In doing so, mutation ensures that no important features are prematurely lost, thus maintaining the mating pool diversity. For this investigation, we have considered reciprocal exchange mutation operator that selects two genes randomly of a chromosome in every cluster and swaps them. The probability of mutation is usually chosen to be considerably less than the probability of crossover. So, mutation plays a secondary role in the GA search. For example, let the chromosome (1, 2, 4, 3, 6, 7, 5) be selected for mutation, and vertices 2 and 4 are swapped in cluster 1, and vertices 7 and 5 are swapped in cluster 2; then the mutated chromosome becomes (1, 4, 2, 3, 6, 5, 7) which is shown in Figure 3. Bold edges in Figure 3(b) are the new edges in the mutated chromosome.

### 3.8. A Local Search Method

We have considered the combined mutation operation as a local search method which has been successfully applied to the bottleneck TSP [8, 22] and maximum TSP [25]. It combines three mutation operators: insertion, inversion, and reciprocal exchange, with cent percentage of probabilities. Insertion operator selects a vertex (gene) in a chromosome and inserts it in a random place, and inversion operator selects two points along the length of a chromosome and reverses the subchromosomes between these points. This local search, a modification of the hybrid mutation operator [26], is applied to a chromosome. Recall that sizes of the clusters *V*_1_, *V*_2_,…, *V*_*m*_ are *n*_1_, *n*_2_,…, *n*_*m*_, respectively. Suppose (1 = *α*_1_, *α*_2_, *α*_3_,…, *α*_*n*_) is a chromosome; then the local search for the OCTSP can be developed as follows.

**Step 0**

Set *x* = 2 and *y* = 1.

**Step 1**

For *i* : = 1 to *m* perform Step 2.

**Step 2**

Set *y* = *n*_*i*_ + *y* and go to Step 3.

**Step 3**

For *j* : = *x* to (*y* − 1) perform Step 4.

**Step 4**

For *k* : = (*j* + 1) to *y* perform Step 5.

**Step 5**

If inserting vertex *α*_*j*_ after vertex *α*_*k*_ reduces the present tour cost, then insert the vertex *α*_*j*_ after vertex *α*_*k*_. In either case go to Step 6.

**Step 6**

If inverting subchromosome between the vertices *α*_*j*_ and *α*_*k*_ reduces the present tour cost, then invert the subchromosome. In either case go to Step 7.

**Step 7**

If swapping the vertices *α*_*j*_ and *α*_*k*_ reduces the present tour cost, then swap them. In either case go to Step 8.

**Step 8**

Set *x* = *y* + 1 and go to Step 1.

### 3.9. Immigration

**Figure 4:**
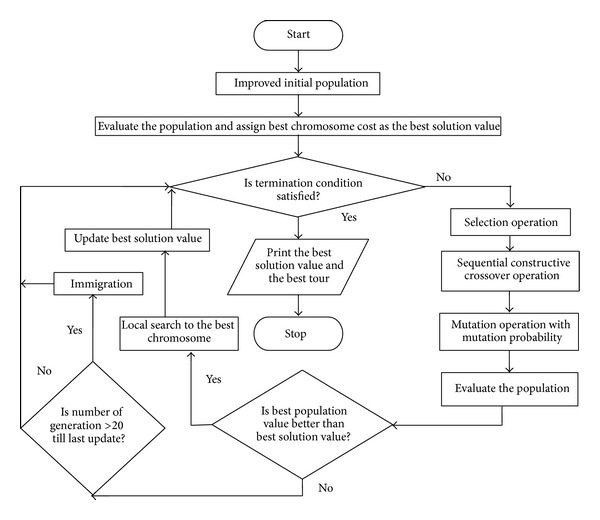
Flowchart of our hybrid genetic algorithm.

It is seen that sometimes GAs get stuck in local minimums for the combinatorial optimization problems, which is very often due to the identical population. So, to improve capability of GAs, the population should be diversified. To diversify the population, immigration method is also adopted, where some randomly selected chromosomes are replaced by new chromosomes after some generations [22]. We are also considering an immigration method. For our investigation, 20% of the population is replaced randomly using sequential sampling algorithm, as discussed in Section 3.4, if no improvement is found within the last 20 generations. Once the immigration is applied, we wait for the next 20 generations for any improvement. Our hybrid GA (HGA) for the OCTSP may be summarized as in Figure 4 [22].

## 4. Results and Discussions

We encoded our HGA in Visual C++, executed on a PC with 3.40 GHz Intel(R) Core (TM) i7-3770 CPU and 8.00 GB RAM under MS Windows 7 operating system, and tested with some TSPLIB [10] instances.

### 4.1. Parameter Setting

GAs are well suited for the combinatorial optimization problems. They find near optimal solution in reasonable time. However, they are guided by suitable choice of parameters, namely, crossover probability (*P*_*c*_), mutation probability (*P*_*m*_), population size (*P*_*s*_), and termination condition. Successful working of GAs depends on a proper selection of these parameters [23]. But, there is not any intelligent rule to set these parameters. In general, various sets of the parameters are tested, and then the best one is selected. We are also following a similar method. So, we set the parameters as follows: a maximum of 20,000 generations as termination condition, 20 as population size, 1.00 (100%) as crossover probability, and 20 independent runs for each setting. However, we are not reporting our experiments except for the mutation probability.

To set mutation probability, six mutation probabilities, 0.00, 0.01, 0.02, 0.03, 0.04, and 0.05, are considered and tested on five asymmetric TSPLIB instances with four clusters (*n*_1_, *n*_2_, *n*_3_, and *n*_4_) for each of the instances ftv110, ftv120, ftv130, ftv140, and ftv150. For example, the 7-vertex instance with two clusters (3, 3) means *V*_1_ = {2,3, 4}, *V*_2_ = {5,6, 7}, and *V*_1_ is followed by *V*_2_.

Table 4 reports the mean and standard deviation (in parenthesis) of the best solution values over 20 trials on five instances, ftv110–ftv150, for different mutation probabilities. The boldface denotes the best average solution value. It is seen that there is significant improvement of the solutions using nonzero mutation probabilities over using zero mutation probability. It shows that mutation operation also plays an important role in GAs. Mutation probabilities 0.03 and 0.04 are competing. Using *P*_*m*_ = 0.03, the algorithm obtains the best average solution for the instances ftv110, ftv120, and ftv150. For the remaining two instances, the algorithm obtains the best average solution at *P*_*m*_ = 0.04. However, if we look at the standard deviation, solutions are relatively stable at *P*_*m*_ = 0.03.

**Figure 5:**
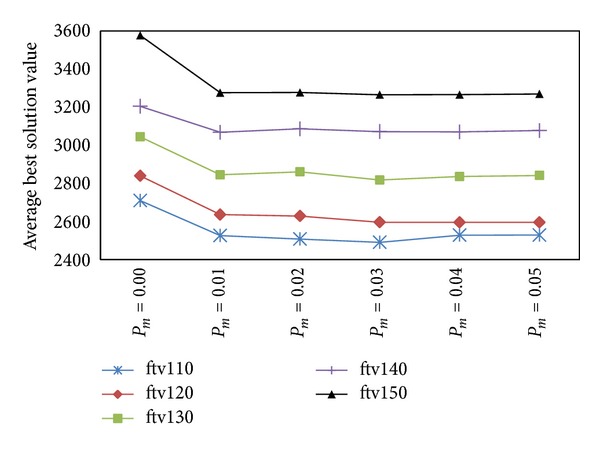
Average best solution values on five asymmetric TSPLIB instances using six mutation probabilities.

Figure 5 plots the average best solution values for the five instances obtained by the HGA using mutation probabilities from 0.00 to 0.05. The figure shows clearly the effectiveness of mutation operator. It is seen that, as mutation probability increases, solution quality also increases. However, after *P*_*m*_ = 0.04, solution quality is not found to be good. From the table and the figure, we can conclude that *P*_*m*_ = 0.03 is suitable for our algorithm. Hence, we are going to use *P*_*m*_ = 0.03 for our further study.

### 4.2. Comparative Study on Asymmetric Instances

We present a comparative study between HGA and LBDCOMP [9] for some asymmetric TSPLIB instances of sizes from 34 to 171 with various clusters and different cluster sizes. It is to be mentioned that LBDCOMP [9] is claimed to find exact optimal solution of the OCTSP instances, which has been disproved by showing results of some small sized instances [11]. Anyway, since no other literature reports the exact solution for large size instances, we are going to compare with the LBDCOMP algorithm to see solution quality by our HGA.  Table 5 shows this comparative study between HGA and LBDCOMP. The table reports results by LBDCOMP, and best solution value (*BestSol*), average solution value (*AvgSol*) in 20 runs, average complete computational time (*CTime*), average computational time when final best solution is seen for the first time (*FTime*) in twenty runs and percentage of error (Error(%)) of the best solution obtained by our HGA. The percentage of error is calculated by the formula

(2) Error(%)=BestSol−OptSolOptSol×100%,

where *BestSol* denotes the best solution obtained by HGA and *OptSol* denotes the solution obtained by LBDCOMP.

It is seen from Table 5 that our HGA finds best/optimal solution of thirty-two instances at least once in twenty runs, whereas LBDCOMP could not find optimal solution for at least six instances—ftv33 with clusters (16, 17) and (9, 24), ftv35 with clusters (17, 18), ftv47 with clusters (13, 34), ftv55 with clusters (27, 28), and ftv170 with clusters (44, 42, 42, 42). That is, for these six instances solution quality by HGA is found to better. On the other hand, for five instances, namely, ftv110, ftv130, ftv140, ftv150, and ftv160, with four clusters each, solution quality by LBDCOMP is better than by our HGA. For these five instances, percentage of error by HGA is at most 0.53%. However, on average, solution quality by HGA is 0.38% better than that of by LBDCOMP.

In terms of computational time, we cannot directly compare the algorithms because they are executed in different machines, and it was not possible to access the original code of LBDCOMP. However, a large gap between computational time by LBDCOMP and HGA is seen in the table, and HGA takes much less time. Further, if *FTime* is considered for HGA, then definitely it is found to be much better than LBDCOMP. It is interesting to see that, for any of these instances with the same number of clusters but different cluster sizes, HGA takes different computational times, and as the size of clusters becomes more unbalanced, computational time increases. In an unbalanced clustered instance, size of the clusters is not equal. It is also seen that, on average, HGA hits final best solution for the first time within 56% of complete computational time. This shows that HGA finds best solution, on average, in the middle of the generations for these asymmetric TSPLIB instances.

### 4.3. Comparative Study on Symmetric Instances

Now we are going to compare our HGA with lexisearch algorithm (LSA) [11] on some small sized symmetric TSPLIB [10] instances with various clusters and different cluster sizes. It is to be noted that our HGA does not require any modification for solving different types and cases of the instances.  Table 6 shows comparative study between LSA and HGA. The solution quality by HGA is found to be insensitive to the number of runs for most of the instances. HGA finds best/optimal solution of twenty-three instances at least once in twenty runs, whereas LSA could not find optimal solution for at least three instances within four hours of computational time, for example, the instances gr48 with clusters (23, 24); and eil51 with clusters (25, 25) and (16, 17, 17). Overall, for these symmetric instances solution quality by HGA is found to be better, and on average, solution quality by HGA is 0.24% better than that by LSA.

In terms of computational time, it can be easily concluded that HGA is much better than LSA, though LSA was executed on slower machine (Pentium IV PC with speed 3 GHz and 448 MB RAM). Of course, the nature of LSA and HGA is not the same; LSA gives exact optimal solution whereas HGA gives heuristic solution. It is also seen from the table that, on average, HGA hits final best solution for the first time within 13% of complete computational time. This shows that HGA finds best solution, on average, in the beginning of the generations for these instances.

### 4.4. Proposed Solution for Some More Symmetric Instances

Table 7 presents results for some more symmetric TSPLIB instances of sizes from 52 to 431 with various clusters and cluster sizes. Since, to the best of our knowledge, no literature presents solution for these instances, hence, we could not provide any comparative study on these instances. However, we present the results for future study of the OCTSP on these instances. For our self-comparison, we provide solution value and percentage of error (in parentheses) by our HGA for the instances with one cluster, which are, of course, usual TSP instances. Out of forty-seven usual TSP instances, HGA finds exact optimal solution to thirty-three instances. For the remaining instances, maximum percentage of error is 1.08%. That means our algorithm can provide near exact solution, if not exact. Treating this study as a base for effectiveness of the algorithm, we can conclude that the reported solutions are near exact solution, if not exact. It is also seen from the table that, for the same instances, as the number of clusters increases solution value also increases. On the other hand, as the number of clusters increases computational time decreases. In general, computational time for solving a single clustered instance (i.e., usual TSP instance) is more than its corresponding multiclustered instances. It seems that the structures of these multiclustered instances are less complex and, hence, easier than their corresponding single clustered instances. For these symmetric instances, on average, HGA hits final best solution for the first time within 43% of complete computational time. This shows that HGA finds best solution for these instances, on average, in the middle of the generations.

## 5. Conclusions

We presented a hybrid genetic algorithm using sequential constructive crossover, 2-opt search, a local search, and an immigration method to obtain heuristic solution to the OCTSP. We have used a sequential sampling method for generating initial population. The efficiency of the hybrid GA to the problem has been examined against the exact partitioning algorithm (LBDCOMP) [9] for some asymmetric TSPLIB instances and the lexisearch algorithm (LSA) [11] for some small sized symmetric TSPLIB instances. The computational experiments show that our HGA is efficient in producing high quality of solution for the benchmark instances. On the basis of solution quality, our HGA is found to be better than the LBDCOMP and LSA. In terms of computational time also, our algorithm is found to be the best one. Finally, we present solution to the problem for some more symmetric TSPLIB instances. Since, to the best of our knowledge, no literature presents solution for these instances, we could not confirm the quality of our solutions for the instances. However, for the symmetric instances of size up to 51, we found that our HGA obtains exact optimal solution to the instances. It is to be noted that HGA does not require any modification for solving different types of TSPLIB instances.

For any instance, as the number of clusters increases the solution value also increases. Computational time for solving a single clustered instance (i.e., usual TSP instance) is more than that for solving its corresponding multiclustered instances. For any multiclustered instance, as the clusters become more unbalanced computational time increases.

## Figures and Tables

**Table 1:** The cost matrix with row-minima and column-minima.

Vertex	1	2	3	4	5	6	7	Row-minima
1	999	75	99	9	35	63	8	8
2	51	999	86	46	88	29	20	20
3	100	5	999	16	28	35	28	5
4	20	45	11	999	59	53	49	11
5	86	63	33	65	999	76	72	33
6	36	53	89	31	21	999	52	21
7	58	31	43	67	52	60	999	32

Column-minima	9	0	0	1	0	9	0	

**Table 2:** The reduced cost matrix.

Vertex	1	2	3	4	5	6	7
1	999*	67	91	0	27	46	0
2	22	999*	66	25	68	0	0
3	86	0	999*	10	23	21	23
4	0	34	0	999*	48	33	38
5	44	30	0	31	999*	34	39
6	6	32	68	9	0	999*	31
7	18	0	12	35	21	29	999*

(*Elements are left as 999).

**Table 3:** The alphabet table.

Vertex	*V*—Cost	*V*—Cost	*V*—Cost	*V*—Cost	*V*—Cost	*V*—Cost	*V*—Cost
1	4—0	7—0	5—27	6—46	2—67	3—91	1—999
2	6—0	7—0	1—22	4—25	3—66	5—68	2—999
3	2—0	4—10	6—21	5—23	7—23	1—86	3—999
4	1—0	3—0	6—33	2—34	7—38	5—48	4—999
5	3—0	2—30	4—31	6—34	7—39	1—44	5—999
6	5—0	1—6	4—9	7—31	2—32	3—68	6—999
7	2—0	3—12	1—18	5—21	6—29	4—35	7—999

**Table 4:** Mean and standard deviation of best solution values on five asymmetric TSPLIB instances.

Instance	Clusters	*P*_*m*_ = 0.00	*P*_*m*_ = 0.01	*P*_*m*_ = 0.02	*P*_*m*_ = 0.03	*P*_*m*_ = 0.04	*P*_*m*_ = 0.05
ftv110	(29, 27, 27, 27)	2709.78 (67.50)	2526.34 (31.84)	2508.00 (25.21)	**2490.80** (23.51)	2528.35 (34.56)	2528.67 (24.79)
ftv120	(30, 30, 30, 30)	2839.67 (82.94)	2636.34 (41.94)	2628.78 (40.89)	2596.23 (30.20)	**2595.68** (35.36)	2595.79 (27.22)
ftv130	(34, 32, 32, 32)	3044.11 (93.77)	2844.89 (55.32)	2860.56 (40.03)	**2817.56** (24.42)	2835.78 (34.23)	2841.66 (19.67)
ftv140	(35, 35, 35, 35)	3204.45 (150.65)	3068.31 (58.07)	3086.29 (54.50)	3071.26 (43.18)	**3070.02** (49.43)	3077.41 (55.58)
ftv150	(39, 37, 37, 37)	3576.25 (154.55)	3275.66 (57.72)	3276.82 (27.14)	**3265.26** (35.60)	3265.55 (29.64)	3269.39 (59.68)

**Table 5:** A comparative study between LBDCOMP and HGA for asymmetric TSPLIB instances.

Instance	Clusters	LBDCOMP		HGA				
		Solution	Time	BestSol	Error (%)	AvgSol	FTime	CTime
ftv33	(16, 17)	1584	5.11	1501	−5.24	1502.16	0.12	1.01
	(9, 24)	1509	5.87	1501	−0.53	1503.24	0.13	1.25
	(3, 30)	1356	5.00	1356	0.00	1359.15	0.18	1.63
ftv35	(17, 18)	1747	11.32	1731	−0.92	1739.29	0.41	1.13
	(10, 25)	1660	3.46	1660	0.00	1663.34	0.32	1.26
	(3, 32)	1527	13.89	1527	0.00	1533.13	0.42	1.41
ftv38	(19, 19)	1681	4.13	1681	0.00	1686.06	0.96	1.35
	(11, 27)	1689	7.65	1689	0.00	1692.25	0.22	1.53
	(3, 35)	1573	17.66	1573	0.00	1586.00	0.16	2.11
ftv44	(22, 22)	1935	24.92	1935	0.00	1940.23	0.17	1.75
	(13, 31)	1830	7.90	1830	0.00	1852.12	0.47	2.01
	(4, 40)	1670	48.71	1670	0.00	1689.00	0.40	2.72
ftv47	(23, 24)	2470	10.06	2470	0.00	2526.24	0.78	1.93
	(13, 34)	2349	10.49	2257	−3.92	2281.27	1.15	2.42
	(4, 43)	1957	5.93	1957	0.00	2006.31	1.52	3.17
ftv55	(27, 28)	2299	6.00	2219	−3.48	2248.24	0.71	2.60
	(16, 39)	1937	8.15	1937	0.00	1982.12	2.07	3.06
	(5, 50)	1763	32.06	1763	0.00	1788.24	1.16	4.48
ftv64	(32, 32)	2658	28.54	2658	0.00	2686.15	2.15	3.68
	(19, 45)	2383	65.27	2383	0.00	2494.11	2.98	4.37
	(6, 58)	2006	110.93	2006	0.00	2048.21	2.24	6.38
ftv70	(35, 35)	2308	135.86	2308	0.00	2341.30	1.30	4.11
	(21, 49)	2244	102.69	2244	0.00	2267.24	2.72	4.79
	(7, 63)	2134	323.31	2134	0.00	2163.32	4.60	7.37
ftv90	(45, 45)	1756	17.71	1756	0.00	1833.78	5.28	6.93
	(27, 63)	1710	56.71	1710	0.00	1784.80	5.55	8.46
	(9, 81)	1579	68.24	1579	0.00	1653.22	5.79	14.43
ftv100	(50, 50)	2008	24.13	2008	0.00	2084.17	4.89	8.90
	(30, 70)	1903	143.51	1903	0.00	1969.66	7.59	11.88
	(10, 90)	1788	187.49	1788	0.00	1904.15	11.72	19.27
ftv110	(29, 27, 27, 27)	2410	289.19	2411	0.04	2490.80	6.08	7.48
ftv120	(30, 30, 30, 30)	2571	83.96	2571	0.00	2596.23	5.66	9.04
ftv130	(34, 32, 32, 32)	2747	331.67	2751	0.15	2817.56	5.95	11.20
ftv140	(35, 35, 35, 35)	2941	571.44	2947	0.20	3071.26	10.02	13.23
ftv150	(39, 37, 37, 37)	3119	81.42	3120	0.03	3265.26	7.94	15.79
ftv160	(40, 40, 40, 40)	3561	754.54	3580	0.53	3696.18	12.51	18.34
ftv170	(44, 42, 42, 42)	3927	297.71	3891	−0.92	3992.01	15.36	22.39

Average			**105.48**		**−0.38**		**3.56**	**6.35**

**Table 6:** A comparative study between LSA and HGA for symmetric TSPLIB instances.

Instance	Clusters	LSA		HGA				
		Solution	Time	BestSol	Error (%)	AvgSol	FTime	CTime
burma14	(6, 7)	3621	0.00	3621	0.00	3621.00	0.00	0.25
ulysses16	(7, 8)	7303	0.00	7303	0.00	7303.00	0.00	0.30
gr17	(8, 8)	2517	0.00	2517	0.00	2517.00	0.00	0.32
gr21	(10, 10)	3465	0.00	3465	0.00	3465.00	0.00	0.48
ulysses22	(10, 11)	8190	0.17	8190	0.00	8190.00	0.00	0.54
gr24	(11, 12)	1558	0.14	1558	0.00	1558.00	0.32	0.63
fri26	(12, 13)	957	0.05	957	0.00	957.00	0.00	0.62
bayg29	(14, 14)	2144	21.03	2144	0.00	2144.00	0.07	0.93
	(9, 9, 10)	2408	35.22	2408	0.00	2408.00	0.00	0.65
bays29	(14, 14)	2702	27.33	2702	0.00	2702.00	0.00	0.94
	(9, 9, 10)	2991	24.89	2991	0.00	2991.00	0.00	0.66
dantzig42	(20, 21)	699	446.56	699	0.00	699.00	0.00	1.51
	(13, 14, 14)	699	1.02	699	0.00	699.00	0.02	1.26
	(10, 10, 10, 11)	699	5.17	699	0.00	699.00	0.00	1.12
swiss42	(20, 21)	1605	14400.00	1605	0.00	1612.33	0.77	2.75
	(13, 14, 14)	1919	14400.00	1919	0.00	1923.00	0.53	1.59
	(10, 10, 10, 11)	1944	14400.00	1944	0.00	1945.42	0.15	1.15
gr48	(23, 24)	6656	14400.00	6433	−3.35	6433.00	0.07	2.01
	(15, 16, 16)	7466	14400.00	7466	0.00	7504.72	0.04	1.58
	(11, 12, 12, 12)	8554	14400.00	8554	0.00	8554.00	0.38	1.43
eil51	(25, 25)	570	14400.00	564	−1.05	564.00	0.73	2.27
	(16, 17, 17)	689	14400.00	681	−1.16	681.00	0.15	1.73
	(12, 12, 13, 13)	714	14400.00	714	0.00	714.00	0.12	1.62

Average			**5659.20**		**−0.24**		**0.15**	**1.15**

**Table 7:** Results on some symmetric TSPLIB instances using HGA.

Instance	Clusters	BestSol	AvgSol	FTime	CTime
berlin52	(51)	7542 (0.00%)	7542.00	0.70	3.63
	(25, 26)	10422	10422.00	0.34	2.50
brazil58	(57)	25395 (0.00%)	25395.00	1.09	4.50
	(28, 29)	34110	34110.00	1.14	3.05
st70	(69)	675 (0.00%)	677.15	1.49	6.15
	(34, 35)	916	916.00	2.05	4.18
eil76	(75)	538 (0.00%)	539.26	2.42	7.33
	(37, 38)	721	723.12	1.99	5.11
pr76	(75)	108159 (0.00%)	108254.55	2.09	7.46
	(37, 38)	120436	120583.13	2.74	5.10
gr96	(95)	55209 (0.00%)	55672.85	5.89	12.07
	(47, 48)	56757	56767.22	3.44	8.40
rat99	(98)	1211 (0.00%)	1218.40	1.16	12.91
	(49, 49)	1346	1348.25	5.53	9.10
kroA100	(99)	21282 (0.00%)	21321.80	3.67	13.02
	(24, 25, 25, 25)	45733	46147.95	3.40	6.77
kroB100	(99)	22141 (0.00%)	22193.15	4.90	13.48
	(24, 25, 25, 25)	45709	45813.85	2.52	7.24
kroC100	(99)	20749 (0.00%)	20789.45	3.04	12.62
	(24, 25, 25, 25)	46388	46475.35	4.10	6.90
kroD100	(99)	21294 (0.00%)	21389.11	4.64	12.26
	(24, 25, 25, 25)	45681	45952.20	3.43	6.07
kroE100	(99)	22068 (0.00%)	22116.39	5.18	14.52
	(24, 25, 25, 25)	45431	45559.25	3.24	7.27
rd100	(99)	7910 (0.00%)	7932.70	4.75	13.46
	(24, 25, 25, 25)	15501	15524.05	3.84	6.34
eil101	(100)	629 (0.00%)	632.75	5.79	16.07
	(25, 25, 25, 25)	1080	1080.00	3.80	8.92
lin105	(104)	14379 (0.00%)	14416.65	5.60	14.39
	(26, 26, 26, 26)	17584	17618.20	2.13	8.25
pr107	(106)	44303 (0.00%)	44405.67	1.77	14.56
	(26, 26, 27, 27)	51487	51538.80	2.14	7.79
gr120	(119)	6942 (0.00%)	6986.95	6.16	20.73
	(29, 30, 30, 30)	13109	13129.15	5.23	10.48
pr124	(123)	59030 (0.00%)	59181.75	3.51	20.77
	(30, 31, 31, 31)	71295	71295.00	1.12	12.38
bier127	(126)	118282 (0.00%)	118419.60	9.33	28.27
	(30, 32, 32, 32)	174112	174250.70	6.83	19.54
ch130	(129)	6110 (0.00%)	6150.50	13.11	30.12
	(32, 32, 32, 33)	12000	12022.05	5.66	20.05
pr136	(135)	96772 (0.00%)	97240.80	14.80	28.36
	(33, 34, 34, 34)	106605	106718.40	8.14	20.04
gr137	(136)	69853 (0.00%)	70429.50	12.00	28.16
	(34, 34, 34, 34)	81628	81715.01	4.22	14.99
pr144	(143)	58537 (0.00%)	58671.19	6.18	30.83
	(35, 36, 36, 36)	69093	69128.34	2.58	20.22
kroA150	(149)	26524 (0.00%)	26629.65	12.33	35.85
	(37, 37, 37, 38)	52824	52988.40	12.74	18.80
kroB150	(149)	26130 (0.00%)	26264.23	17.21	38.07
	(37, 37, 37, 38)	54008	54237.75	13.15	19.16
ch150	(149)	6528 (0.00%)	6556.31	20.92	38.59
	(37, 37, 37, 38)	13042	13085.25	10.20	19.04
pr152	(151)	73682 (0.00%)	74017.45	11.92	34.25
	(37, 38, 38, 38)	79941	79941.00	1.80	24.28
u159	(158)	42080 (0.00%)	42336.10	12.85	38.87
	(39, 39, 40, 40)	42287	42302.45	3.29	20.94
si175	(174)	21407 (0.00%)	21412.10	51.19	95.68
	(43, 43, 44, 44)	22893	22910.65	7.61	34.72
brg180	(179)	1950 (0.00%)	2010.35	14.29	44.58
	(44, 45, 45, 45)	19430	21060.20	6.90	15.29
rat195	(194)	2323 (0.00%)	2362.20	27.13	71.36
	(48, 48, 49, 49)	2544	2551.72	20.86	35.24
d198	(197)	15800 (0.13%)	15858.75	24.60	77.79
	(49, 49, 49, 50)	17320	17339.50	29.76	43.92
kroA200	(199)	29420 (0.18%)	29618.80	21.08	70.41
	(49, 50, 50, 50)	62514	62941.75	23.45	39.81
kroB200	(199)	29463 (0.09%)	29807.00	23.87	75.01
	(49, 50, 50, 50)	62842	63253.11	18.28	41.40
gr202	(201)	40160 (0.00%)	40413.85	36.96	97.25
	(50, 50, 50, 51)	44176	44248.20	24.86	40.43
ts225	(224)	126643 (0.00%)	127006.83	28.51	93.85
	(56, 56, 56, 56)	171269	171543.30	25.73	48.77
tsp225	(224)	3923 (0.18%)	3967.35	38.29	105.30
	(56, 56, 56, 56)	5133	5171.15	20.11	54.91
pr226	(225)	80467 (0.12%)	80953.60	16.28	87.69
	(56, 56, 56, 57)	96508	96510.10	28.38	63.22
gr229	(228)	134957 (0.26%)	136184.35	37.33	119.79
	(57, 57, 57, 57)	143028	143632.45	32.04	57.31
gil262	(261)	2391 (0.55%)	2403.15	53.08	151.26
	(65, 65, 65, 66)	4874	4906.45	64.33	93.72
pr264	(263)	49219 (0.17%)	49814.45	48.75	149.99
	(65, 66, 66, 66)	60161	60294.15	22.78	97.20
a280	(279)	2585 (0.23%)	2614.05	105.25	187.03
	(69, 70, 70, 70)	2740	2743.75	32.75	94.28
pr299	(298)	48375 (0.38%)	48857.06	38.11	202.84
	(74, 74, 75, 75)	55253	55951.65	45.36	123.61
lin318	(317)	42301 (0.65%)	42679.65	85.68	253.92
	(79, 79, 79, 80)	52578	52811.35	62.29	133.35
rd400	(399)	15370 (0.58%)	15452.20	203.27	546.74
	(99, 100, 100, 100)	30821	31006.60	151.40	259.78
fl417	(416)	11930 (0.58%)	12004.63	227.38	544.17
	(104, 104, 104, 104)	20457	20576.24	201.62	346.01
gr431	(430)	173270 (1.08%)	176047.20	300.35	716.64
	(107, 107, 108, 108)	185162	186661.56	291.81	406.47

Average				**29.86**	**70.16**

## References

[1] Chisman JA (1975). The clustered traveling salesman problem. *Computers and Operations Research*.

[2] Gendreau M, Hertz A, Laporte G (1996). The traveling salesman problem with backhauls. *Computers and Operations Research*.

[3] Guttmann-Beck N, Hassin R, Khuller S, Raghavachari B (2000). Approximation algorithms with bounded performance guarantees for the clustered traveling salesman problem. *Algorithmica*.

[4] Lokin FCJ (1979). Procedures for travelling salesman problems with additional constraints. *European Journal of Operational Research*.

[5] Laporte G, Potvin J-Y, Quilleret F (1997). Tabu search heuristic using genetic diversification for the clustered traveling salesman problem. *Journal of Heuristics*.

[6] Laporte G, Palekar U (2002). Some applications of the clustered travelling salesman problem. *Journal of the Operational Research Society*.

[7] Ahmed ZH (2010). Genetic algorithm for the traveling salesman problem using sequential constructive crossover operator. *International Journal of Biometrics & Bioinformatics*.

[8] Ahmed ZH (2010). A hybrid sequential constructive sampling algorithm for the bottleneck traveling salesman problem. *International Journal of Computational Intelligence Research*.

[9] Aramgiatisiris T (2004). An exact decomposition algorithm for the traveling salesman problem with backhauls. *Journal of Research in Engineering and Technology*.

[10] TSPLIB http://comopt.ifi.uni-heidelberg.de/software/TSPLIB95/.

[11] Ahmed ZH (2013). An exact algorithm for the clustered traveling salesman problem. *Opsearch*.

[12] Little JDE, Murthy KG, Sweeny DW, Karel C (1963). An algorithm for the travelling salesman problem. *Operations Research*.

[13] Jongens K, Volgenant T (1985). The symmetric clustered traveling salesman problem. *European Journal of Operational Research*.

[14] Gendreau M, Laporte G, Potvin JY (1994). Heuristics for the clustered traveling salesman problem.

[15] Potvin J-Y, Guertin F (1995). A genetic algorithm for the clustered traveling salesman problem with an a priori order on the clusters.

[16] Potvin J-Y, Guertin F, Osman IH, Kelly J (1996). The clustered traveling salesman problem: a genetic approach. *Meta-Heuristics: Theory & Applications*.

[17] Anily S, Bramel J, Hertz A (1999). 5/3-Approximation algorithm for the clustered traveling salesman tour and path problems. *Operations Research Letters*.

[18] Christofides N (1976). Worst-case analysis of a new heuristic for the traveling salesman problem.

[19] Sheng W, Xi N, Song M, Chen Y (2005). Robot path planning for dimensional measurement in automotive manufacturing. *Journal of Manufacturing Science and Engineering, Transactions of the ASME*.

[20] Ding C, Cheng Y, He M (2007). Two-level genetic algorithm for clustered traveling salesman problem with application in large-scale TSPs. *Tsinghua Science and Technology*.

[21] Goldberg DE (1989). *Genetic Algorithms in Search, Optimization and Machine Learning*.

[22] Ahmed ZH (2013). A hybrid genetic algorithm for the bottleneck traveling salesman problem. *ACM Transactions on Embedded Computing Systems*.

[23] Deb K (1995). *Optimization for Engineering Design: Algorithms and Examples*.

[24] Ahmed ZH (2011). Multi-parent extension of sequential constructive crossover for the travelling salesman problem. *International Journal of Operational Research*.

[25] Ahmed ZH (2013). An experimental study of a hybrid genetic algorithm for the maximum travelling salesman problem. *Mathematical Sciences*.

[26] Wang C-X, Cui D-W, Wang Z-R, Chen D A novel ant colony system based on minimum 1-tree and hybrid mutation for TSP.

